# Tetraphenylethene-Based Cross-Linked Conjugated Polymer Nanoparticles for Efficient Detection of 2,4,6-Trinitrophenol in Aqueous Phase

**DOI:** 10.3390/ma16196458

**Published:** 2023-09-28

**Authors:** Shengjie Li, Tianwen Ouyang, Xue Guo, Wenyue Dong, Zhihua Ma, Teng Fei

**Affiliations:** 1 School of Materials Science and Engineering, Changchun University of Science and Technology, Changchun 130022, China; 2 Chongqing Research Institute, Changchun University of Science and Technology, Chongqing 401135, China; 3 State Key Laboratory of Integrated Optoelectronics, College of Electronic Science and Engineering, Jilin University, Changchun 130012, China

**Keywords:** fluorescence sensing, explosives detection, polymer nanoparticles, tetraphenylethene

## Abstract

The cross-linked conjugated polymer poly(tetraphenylethene-*co*-biphenyl) (PTPEBP) nanoparticles were prepared by Suzuki-miniemulsion polymerization. The structure, morphology, and pore characteristics of PTPEBP nanoparticles were characterized by FTIR, NMR, SEM, and nitrogen adsorption and desorption measurements. PTPEBP presents a spherical nanoparticle morphology with a particle size of 56 nm; the specific surface area is 69.1 m^2^/g, and the distribution of the pore size is centered at about 2.5 nm. Due to the introduction of the tetraphenylethene unit, the fluorescence quantum yield of the PTPEBP nanoparticles reaches 8.14% in aqueous dispersion. Combining the porosity and nanoparticle morphology, the fluorescence sensing detection toward nitroaromatic explosives in the pure aqueous phase has been realized. The Stern–Volmer quenching constant for 2,4,6-trinitrophenol (TNP) detection is 2.50 × 10^4^ M^−1^, the limit of detection is 1.07 μM, and the limit of quantification is 3.57 μM. Importantly, the detection effect of PTPEBP nanoparticles toward TNP did not change significantly after adding other nitroaromatic compounds, indicating that the anti-interference and selectivity for TNP detection in aqueous media is remarkable. In addition, the spike recovery test demonstrates the potential of PTPEBP nanoparticles for detecting TNP in natural environmental water samples.

## 1. Introduction

A typical nitroaromatic compound, 2,4,6-Trinitrophenol (TNP) is one of the most widely used explosives [1,2,3]. Because of its highly explosive power and easy synthesis, TNP poses a serious threat to social security and the safety of human life and property [4,5,6]. In addition, TNP is also commonly used in the chemical industry and can pollute groundwater and soil due to its toxicity [7,8]. Therefore, finding a safe and reliable way to detect TNP, especially in the aqueous phase, is of great significance. At present, there are many methods developed for TNP detection, such as nuclear magnetic resonance (NMR) [9], energy dispersive X-ray diffraction [10], mass spectrometry [11], electrochemical methods [12], fluorescence sensing [13,14,15], and others. Among these, fluorescence sensing has been widely used in the detection of TNP because of its advantages of high sensitivity, selectivity, and low cost [16,17].

Numerous materials have been developed for fluorescence sensing, such as metal organic frameworks (MOFs) [18,19], conjugated polymers [20,21], quantum dots [22,23,24,25], fluorescent cross-linked conjugated polymers [26,27,28,29], and others. Fluorescent cross-linked conjugated polymers offer many advantages, such as high specific surface area [30], inherent porosity [31], structural flexibility [32], chemical stability [33], and low density [34]; however, the traditional fluorescent cross-linked conjugated polymers suffer aggregation-caused quenching (ACQ), caused by the intensive π–π interactions in the aggregated state, which is detrimental to the application in fluorescence sensing. Tetraphenylethene with the peripheral phenyl rotors connected to the double bond can effectively suppress the aggregation and lead to a good fluorescence quantum yield in the aggregated state [35]. Various fluorescent materials, including the cross-linked conjugated polymers constructed from tetraphenylethene, are widely synthesized and used in the field of organic optoelectronics, especially in the application of fluorescence sensing. Nevertheless, the highly cross-linked chemical network structures make the cross-linked conjugated polymers insoluble or not finely dispersed in water, thus limiting the application of fluorescence sensing in the aqueous phase.

In this manuscript, cross-linked conjugated polymer poly(tetraphenylethene-*co*-biphenyl) (PTPEBP) nanoparticles were synthesized based on tetraphenylethene structure by Suzuki coupling in water/toluene miniemulsion. PTPEBP possesses nano-spherical morphology and porous structure, with a particle size of about 56 nm, BET surface area of 69.1 m^2^/g, and a pore diameter concentrated at 2.5 nm. The nano-spherical morphology and high porosity make PTPEBP nanoparticles viable candidates for nitroaromatics detection in aqueous media. The fluorescence titration experiments indicate the good sensing property of PTPEBP nanoparticles toward nitroaromatics in water, especially for TNP detection with a Stern–Volmer quenching constant of 2.50 × 10^4^ M^−1^. For comparison, some literature data on polymer-based fluorescence sensing materials for TNP detection are summarized in Table 1, indicating that PTPEBP nanoparticles are very promising for fluorescence sensing toward TNP detection in the aqueous phase. Moreover, selective and anti-interference detection of TNP with the presence of other nitroaromatics and common ions in water was achieved. The spike recovery tests in natural environmental water samples demonstrate a high-performance detection toward TNP based on PTPEBP nanoparticles at a 95% confidence level (*t*-test).

## 2. Experiment

### 2.1. Reagents and Measurements

All details for reagents and measurements can be found in the Supporting Information.

### 2.2. Synthesis

Sodium dodecyl sulfate (SDS, 0.20 g, 0.69 mmol) and potassium carbonate (K_2_CO_3_) (0.25 g, 1.80 mmol) were dissolved in deionized water (20 mL) and ultrasonicated for 10–15 min. The components of [1,1′-biphenyl]-4,4′-diyldiboronic acid (0.07 g, 0.30 mmol), 1,1,2,2-tetrakis(4-bromophenyl)ethene (0.10 g, 0.15 mmol), and tetrakis(triphenylphosphine)palladium (Pd(PPh_3_)_4_) (0.01 g, 0.01 mmol) were dissolved in toluene (2 mL). The aqueous phase was then combined with the organic phase, and the mixture was ultrasonicated for 10 min to conduct a stable miniemulsion. After ultrasonication, the prepared miniemulsion was heated to 85 °C for 20 h under a nitrogen atmosphere. After the reaction, the miniemulsion was stirred in the air to evaporate the residual toluene, and the solution was dialyzed (Mn 3500 g/mol) in deionized water to remove the surfactant, unreacted monomers, and low molecular weight oligomers. The product was collected by centrifugation (10,000 rpm, 20 min) and dried to obtain cross-linked conjugated polymer PTPEBP nanoparticles. Yield: 0.035 g (37%).

## 3. Results and Discussion

### 3.1. Synthesis and Characterization

**Scheme 1:**
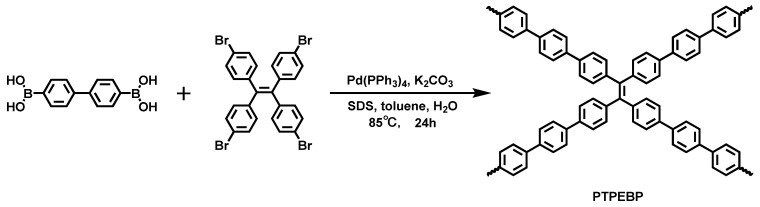
Synthetic route of PTPEBP nanoparticles.

Scheme 1 exhibits the synthetic route for PTPEBP nanoparticles.

**Figure 1:**
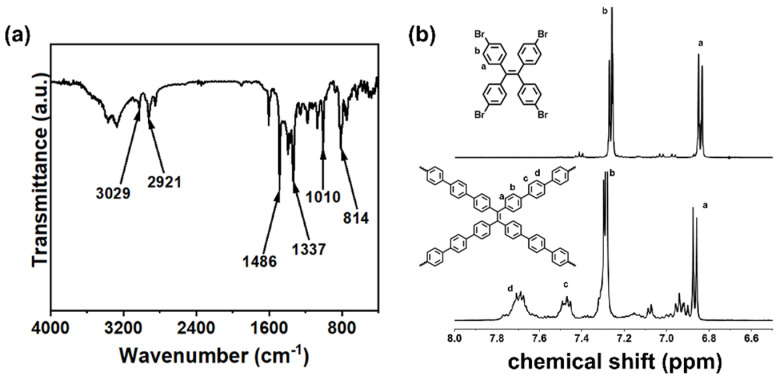
(**a**) FTIR spectrum of PTPEBP; (**b**) ^1^H NMR spectra of PTPEBP and 1,1,2,2-tetrakis(4-bromophenyl)ethylene in CDCl_3_.

The monomer 1,1,2,2-tetrakis(4-bromophenyl)ethene was synthesized according to the literature [48]. The polymer PTPEBP nanoparticles were prepared by the Suzuki-miniemulsion polymerization method. The structure of PTPEBP was characterized by Fourier transform infrared spectroscopy (FTIR) and ^1^H nuclear magnetic resonance (^1^H NMR). As shown in Figure 1a, the peaks near 3029 and 2921 cm^−1^ are attributed to the C–H stretching vibration on the phenyl ring of PTPEBP. The peaks at 1486 and 1337 cm^−1^ are the stretching vibrations of the carbon skeleton on the phenyl ring. The peaks at 1010 and 814 cm^−1^ represent the C–H bending vibration on the phenyl ring. As shown in Figure S1, the C–Br vibrational peak of the monomer 1,1,2,2-tetrakis(4-bromophenyl)ethylene at 503 cm^−1^ has completely disappeared, indicating the success of Suzuki coupling.

The ^1^H NMR spectra of the polymer PTPEBP nanoparticles and the corresponding monomer 1,1,2,2-tetrakis(4-bromophenyl)ethylene are shown in Figure 1b. Compared with 1,1,2,2-tetrakis(4-bromophenyl)ethylene, the signal peaks of the polymer PTPEBP are complicated and shifted with the occurrence of new signals due to the highly cross-linked structure, indicating the successful synthesis of the polymer PTPEBP. The molecular weight of PTPEBP nanoparticles was measured through the static light scattering (SLS) method, and the number-average molecular weight (Mn) is 6.618 × 10^4^ g/mol with the polydispersity (PDI) of 3.218 (Table S2).

### 3.2. Morphology and Porosity 

**Figure 2:**
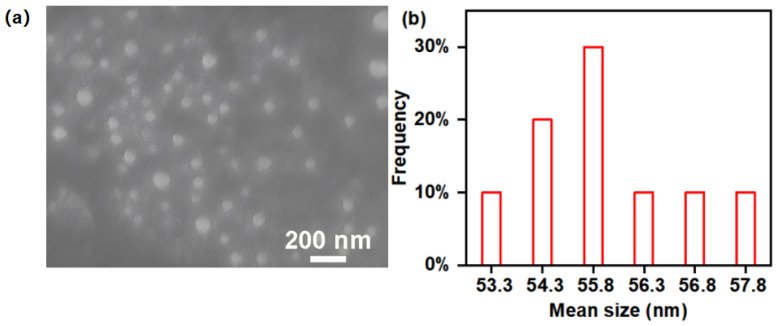
(**a**) SEM image; (**b**) particle size distribution of PTPEBP.

The characteristics of morphology and pore of the polymer PTPEBP were characterized by scanning electron microscopy (SEM) and nitrogen isothermal adsorption-desorption measurements. As shown in Figure 2, it can be seen from the SEM image that the polymer PTPEBP exhibits a spherical shape and uniform particle size with an average particle size of about 56 nm, confirming the successful preparation of polymer PTPEBP nanoparticles.

Figure S2 shows the N_2_ adsorption-desorption curve and the distribution of pore size of the PTPEBP. PTPEBP nanoparticles exhibit the characteristics of a type IV isotherm in the range of P/P_0_ = 0.02–1. The BET surface area is 69.1 m^2^/g, and the pore diameter is concentrated at ca. 2.5 nm. The nanoparticle morphology and porosity of the cross-linked polymer PTPEBP could promote its dispersion in water and provide channels for the diffusion and adsorption of explosive molecules, which are conducive to improving the fluorescence sensing performance of explosive detection in aqueous media.

### 3.3. Photophysical Property

**Figure 3:**
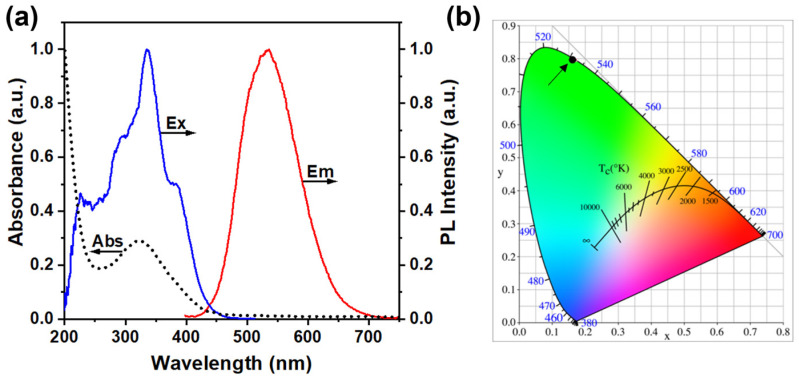
(**a**) UV–Vis absorption, excitation, and fluorescence emission spectra of PTPEBP nanoparticles in pure aqueous phase; (**b**) CIE chromaticity diagram of the emission spectrum.

As shown in Figure 3a, the UV–Vis absorption, excitation, and fluorescence emission spectra of polymer PTPEBP nanoparticles in deionized water were tested. The detailed photophysical data are given in Table 2.

An absorption peak at 320 nm was observed in the absorption spectrum, which is attributed to the characteristic peak generated by the π–π* transition of the polymer backbone. The strongest excitation wavelength in the excitation spectrum is 336 nm, with a shoulder at 375 nm. In the fluorescence spectrum, the maximum emission wavelength of PTPEBP is 532 nm with a peak width of 110 nm at half-height, which is the green emission (Figure 3b). Taking quinine sulfate as a reference, the fluorescence quantum yield (PLQY) of PTPEBP nanoparticles in pure aqueous dispersion was measured to be 8.14%. 

### 3.4. Detection of Nitroaromatic Explosives in the Aqueous Phase

**Figure 4:**
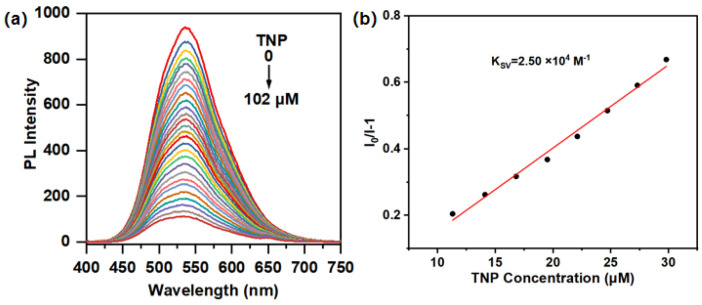
(**a**) Fluorescence quenching spectra of PTPEBP nanoparticles in aqueous dispersion upon TNP addition; (**b**) Stern–Volmer plot of relative fluorescence intensity of PTPEBP nanoparticles vs. TNP concentration.

PTPEBP nanoparticles have a large specific surface area and good porosity, which provide the possibility for sensitive detection of nitroaromatic explosives in the aqueous phase. TNP, 1,3,5-trinitrobenzene (TNB), and 2,4-dinitrotoluene (DNT) were chosen as model explosives for investigation. The fluorescence spectra of PTPEBP nanoparticles in the presence of TNP in aqueous media are shown in Figure 4a.

It is obviously demonstrated that the fluorescence intensity of polymer PTPEBP nanoparticles weakens by degrees as TNP concentration increases. When the concentration of TNP reaches 102 μM, the degree of fluorescence quenching reaches 90%, demonstrating the good capacity of PTPEBP nanoparticles for TNP sensing. The Stern–Volmer plot of the relative fluorescence intensity of the polymer PTPEBP nanoparticles vs. the TNP concentration is shown in Figure 4b to explore the detection sensitivity of the sensing material. In the TNP concentration range of 10–30 μM, the Stern–Volmer curve can be fitted linearly, and the Stern–Volmer quenching constant (K_SV_) of PTPEBP nanoparticles for TNP in aqueous phase was calculated to be 2.50 × 10^4^ M^−1^. Measuring the initial fluorescence intensity of PTPEBP nanoparticles in aqueous dispersion multiple times determined the standard deviation σ to be 4.93. Moreover, the slope K was determined to be 1.38 × 10^7^ M^−1^. This finding was obtained by the linear fitting of the fluorescence intensity of polymer PTPEBP vs. the TNP concentration (Figure S3). On the basis of the formula LOD = 3σ/K and LOQ = 10σ/K, the limit of determination (LOD) of PTPEBP nanoparticles for detecting TNP is 1.07 μM, and the limit of quantitation (LOQ) is 3.57 μM, indicating that the polymer PTPEBP nanoparticles have good fluorescence sensing performance for TNP detection. 

**Figure 5:**
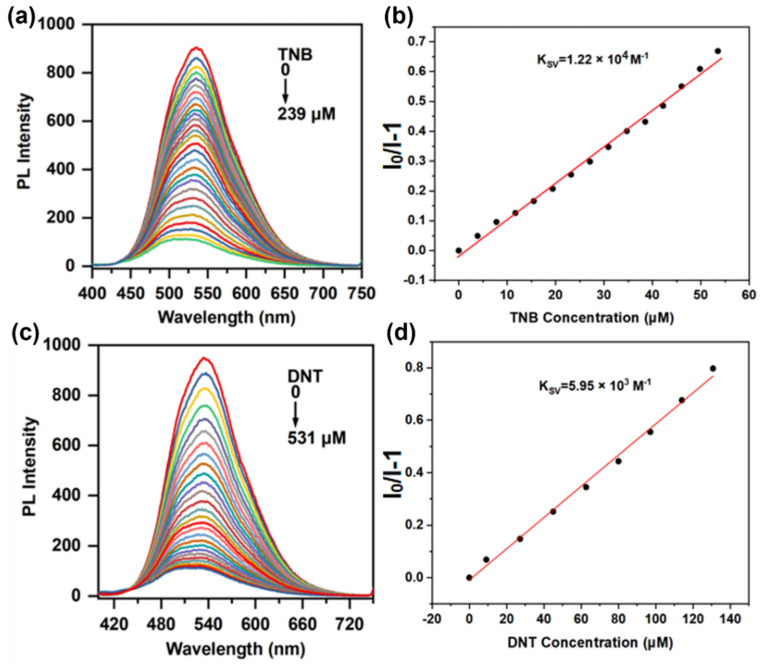
Fluorescence quenching spectra of PTPEBP nanoparticles in aqueous media upon TNB (**a**) and DNT (**c**) addition; Stern–Volmer plot of relative fluorescence intensity of PTPEBP nanoparticles of TNB (**b**) and DNT (**d**) concentration.

The fluorescence quenching spectra of PTPEBP nanoparticles in aqueous dispersion for TNB and DNT sensing are shown in Figure 5.

When the concentrations of TNB and DNT increase, the fluorescence intensity of PTPEBP nanoparticles also gradually decreases. However, the quenching effect of PTPEBP nanoparticles was weaker than that of TNP. The concentrations of TNB and DNT as high as 239 μM and 531 μM, respectively, are required to achieve 90% fluorescence quenching of PTPEBP nanoparticles. As shown in Figure 5b, in the concentration range of 0–55 μM TNB, the K_SV_ of PTPEBP nanoparticles for TNB detection is 1.22 × 10^4^ M^−1^, which is lower than TNP. The LOD and LOQ of PTPEBP nanoparticles toward TNB were determined to be 1.81 μM and 6.05 μM, respectively (Figure S4). Through the Stern–Volmer plot of polymer PTPEBP nanoparticles toward DNT in Figure 5d, the K_SV_ obtained was 5.95 × 10^3^ M^−1^, which is 10 times lower than that of TNP. The LOD for DNT is 3.91 μM, and the LOQ is 13.04 μM (Figure S5), which are much higher than those of TNP. It can be seen from the results that the polymer PTPEBP nanoparticles have the best detection effect toward TNP; therefore, TNP was chosen for the selectivity and ion anti-interference tests of polymer PTPEBP nanoparticles in the aqueous phase.

### 3.5. Selectivity of TNP Detection

**Figure 6:**
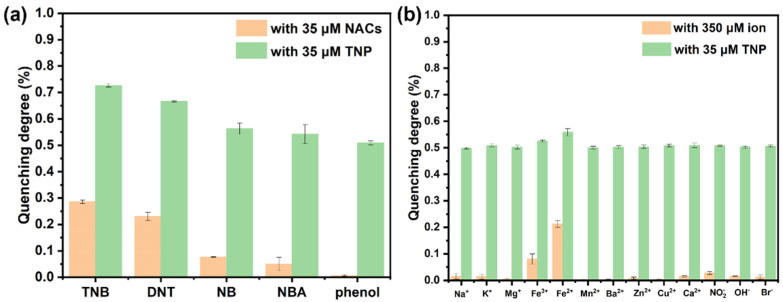
(**a**) Selectivity of PTPEBP nanoparticles for TNP detection; (**b**) ion interference test chart.

In actual application, it is recommended to implement the selective detection of nitroaromatics in aqueous media. As shown in Figure 6a, the detection effects toward TNP in the presence of TNB, DNT, nitrobenzene (NB), 4-nitrobenzoic acid (NBA), and phenol (for comparison) in water were assessed.

When 35 μM of TNB, DNT, NB, NBA, and phenol are added, the quenching degrees of PTPEBP nanoparticles are low; among these, the quenching efficiencies toward DNT and TNB are relatively high, with only about 20%. After the same concentration of TNP is added, the fluorescence intensity of PTPEBP nanoparticles is obviously quenched, and the quenching degree is higher than 50%, which is equivalent to the sensing performance of adding TNP alone. The result indicates good selectivity for TNP detection in the aqueous phase based on PTPEBP nanoparticles. In the actual environmental water sample, the presence of various ions may affect the fluorescence detection result; thus, the anti-interference of the sensing materials to common ions in water is especially important. Figure 6b shows the fluorescence detection performance of PTPEBP nanoparticles toward TNP in the presence of various ions in the aqueous phase. First, 350 μM of common metal cations, halide ions, and other anions were added to PTPEBP aqueous dispersions. It was found that the ions except Fe^2+^ caused very little effect on the fluorescence intensity of the polymer; then, adding 35 μM TNP, the fluorescence intensity decreased significantly, and the quenching degree reached about 50%. Therefore, the influence of ions on PTPEBP in the environment is almost negligible, demonstrating the good ion anti-interference of PTPEBP nanoparticles for TNP detection in natural water samples.

### 3.6. Application

Based on the sensitivity, selectivity, and ion interference in the detection of TNP in the aqueous phase, the standard spike recovery method was used to evaluate the feasibility of polymer PTPEBP nanoparticles for the application of TNP detection in natural environmental water samples. Three water samples from diverse sources were chosen: deionized water, mineral water, and tap water. Next, a spike amount of 5.00 mg/L was added. The spiked equation is Y = 0.13705X − 0.24759 (R^2^ = 0.99246), which was calculated based on the linear fitting curve of the relative fluorescence intensity of PTPEBP nanoparticles vs. the corresponding TNP, as shown in Figure S6. It is shown in Table 3 that the relative standard deviations of PTPEBP nanoparticles to TNP in the three water samples are 2.04%, 1.16%, and 2.18%, respectively. The recoveries are 98.00%, 99.37%, and 101.32%, respectively, which are very similar to those of the high-performance liquid chromatography (HPLC) method [36].

Moreover, the *t*-test method was used to verify the spike recovery tests, as shown in Table 4. The results show the acceptable analysis with t_ref._ values less than 4.30 at a 95% confidence level, thus demonstrating the reliable detection of TNP in real water samples with high sensitivity and selectivity by the fluorescence sensing method using the polymer PTPEBP nanoparticles as sensing material. 

**Figure 7:**
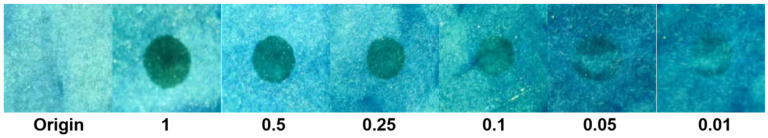
Fluorescence quenching of the PTPEBP test strips within different TNP concentrations (0, 1, 0.5, 0.25, 0.1, 0.05, 0.01 mg/mL) (under 365 nm excitation).

To realize the rapid on-site detection of TNP, the PTPEBP solution was dropped on the filter paper, and the PTPEBP test paper was prepared after drying. As shown in Figure 7, the original PTPEBP paper exhibits a green emission under 365 nm ultraviolet light.

When 10 μL of different concentrations of TNP solution were dropped on the test paper, the visible fluorescence quenching could be noticed with the naked eye, with the recognized TNP concentration of 0.01 mg/mL. The result demonstrates that the polymer PTPEBP nanoparticles could detect TNP as a solid sensor in the practical application.

### 3.7. Fluorescence Sensing Mechanism

**Figure 8:**
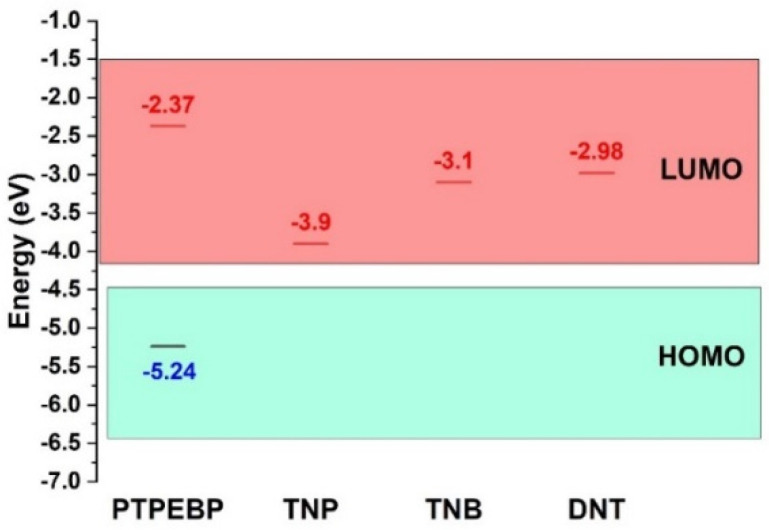
Energy level diagram of PTPEBP, TNP, TNB, and DNT.

The polymer PTPEBP nanoparticles exhibit remarkable sensitivity and selectivity toward TNP detection in aqueous media, benefiting from the nano-spherical morphology and porosity. To better understand the quenching performance toward TNP, the sensing mechanism was investigated in detail. Figure S7 shows the cyclic voltammetry (CV) curve of PTPEBP nanoparticles. According to the CV curve, the initial oxidation potential of PTPEBP nanoparticles is 0.88 V, which is attributed to the oxidation of the polymer backbone. According to the formula, $\mathrm{HOMO}=-\left(E_{\mathrm{Ox}}^{\mathrm{onset}}+4.39\right)\;eV$, the HOMO energy level was determined to be −5.24 eV. Combining with the E_g_ of 2.87 eV calculated by the UV–Vis absorption spectrum of the polymer aqueous dispersion through E_g_ = 1240/λ_g_ eV, the LUMO energy level was determined to be −2.37 eV. Comparing the LUMO energy level of PTPEBP nanoparticles to that of the nitroaromatic explosives [49,50], as shown in Figure 8, the LUMO energy level of PTPEBP nanoparticles is significantly higher than that of TNP, TNB, and DNT, thus a photo-induced excited state electron transfer process may exist in the process of fluorescence quenching [51,52]. The LUMO energy level of TNP is the lowest among these explosives, which could be one reason for the higher detection sensitivity of PTPEBP nanoparticles toward TNP.

**Figure 9:**
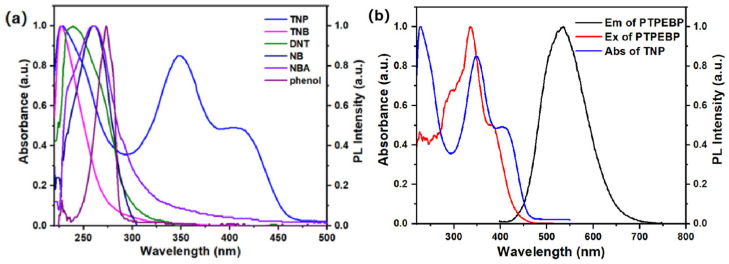
(**a**) UV–Vis absorption spectra of nitro explosives in the aqueous phase; (**b**) excitation and fluorescence spectra of PTPEBP and UV–Vis absorption spectrum of TNP in the aqueous phase.

The excitation and fluorescence spectra of PTPEBP nanoparticles and UV–Vis absorption spectra of various nitroaromatic explosives were tested in water. As we can see in Figure 9b, the absorption spectrum of TNP and the fluorescence spectrum of PTPEBP nanoparticles have a small overlap; thus, Förster resonance energy transfer might occur. In addition, the absorption spectrum of TNP overlaps with the excitation spectrum of PTPEBP nanoparticles to an extensive range, so there may be an inner filter effect in the process of TNP fluorescence quenching.

**Figure 10:**
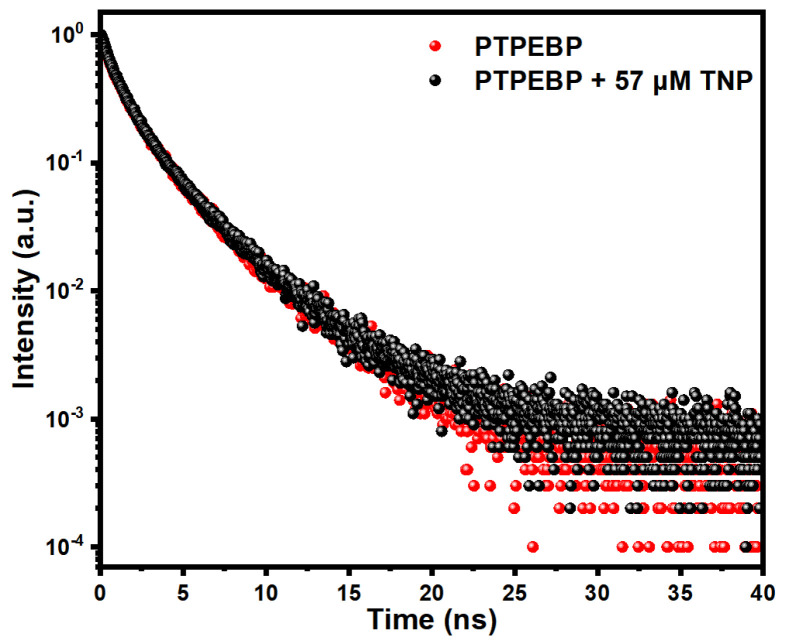
Fluorescence lifetime curves of PTPEBP nanoparticles before and after TNP addition.

In addition, to further acquaint the sensing mechanism, the fluorescence lifetime of PTPEBP nanoparticles in aqueous media before and after adding TNP was evaluated by time-resolved fluorescence spectroscopy. As shown in Figure 10, the fitted fluorescence lifetime curve indicates that the average lifetime of the polymer PTPEBP nanoparticles is 2.20 ns.

After adding TNP, the fluorescence lifetime is 2.23 ns. Almost no apparent change can be found in the lifetimes of PTPEBP nanoparticles before and after TNP addition, which demonstrates that the fluorescence inner filter effect is the foremost mechanism for the detection of TNP in the aqueous phase rather than photo-induced electron transfer and Förster resonance energy transfer.

## 4. Conclusions

The cross-linked conjugated polymer PTPEBP nanoparticles containing tetraphenylethene units were synthesized by Suzuki coupling polymerization in water/toluene miniemulsion, and the structure, morphology, and pore characteristics of the polymer PTPEBP were characterized by FTIR, NMR, SEM, and N_2_ adsorption/desorption experiments, respectively. The obtained PTPEBP exhibits a nano-spherical morphology and porosity, with a particle size of ca. 56 nm and pore size of ca. 2.5 nm. Combining the porosity of the polymer and the characteristics of nanoparticles, the fluorescence sensing property toward nitroaromatic explosives in pure water was conducted. Eventually, the sensitive, selective, and ion anti-interference detection of polymer PTPEBP nanoparticles for TNP was realized, with K_SV_ of 2.50 × 10^4^ M^−1^ and LOD of 1.07 μM. The spike recovery tests indicate no considerable difference between the fluorescence sensing method based on PTPEBP nanoparticles and HPLC analysis for TNP detection in natural environmental water samples at a 95% confidence level. By exploring the fluorescence quenching mechanism, it is concluded that the sensitive and selective detection of TNP by PTPEBP nanoparticles in the aqueous phase is mainly due to the fluorescence inner filter effect.

## Figures and Tables

**Table 1:** Fluorescence quenching data for TNP in water phase from the literature.

Polymeric Material	K_sv_ (M^−1^)	LOD	Ref.
PTPEBP	2.50 × 10^4^	1.07 μM	This Work
PTATE2	1.8 × 10^4^	722 nM	[36]
PTATE4	2.4 × 10^4^	564 nM	[36]
P1	405.79 × 10^4^	0.174 μM	[37]
P2	89.16 × 10^4^	0.511 μM	[37]
P3	40.25 × 10^4^	0.355 μM	[37]
P3*	42.36 × 10^4^	1.18 μM	[37]
P4	54.21 × 10^4^	0.478 μM	[37]
P5	76.24 × 10^4^	0.174 μM	[37]
PTPA-TPE	3.2 × 10^4^	316 nM	[38]
PTPA-TPE2	2.8 × 10^4^	485 nM	[38]
P(PySt-EGDMA)	2.4 × 10^4^	0.329 μM	[39]
oTPETP	1.64 × 10^2^	0.053 mM	[40]
P1e2c	1.72 × 10^4^	510 nM	[41]
PBEMA	2.45 × 10^5^	0.11 μM	[42]
SM-1	1.1 × 10^5^	1.9 μM	[43]
SM-2	5.3 × 10^4^	93 ppb	[43]
BTPC	1.93 × 10^4^	370 nM	[44]
APPTPEP	2.30 × 10^6^	31.5 nM	[45]
CP1	2.16 × 10^4^	28 ppb	[46]
CP2	1.52 × 10^4^	14 ppb	[46]
PMTPBA	7.15 × 10^5^	5.0 × 10^−8^ M	[47]

**Table 2:** Summary of photophysical properties of PTPEPB.

	λ_Abs_ (nm)	λ_Ex_ (nm)	λ_Em_ (nm)	FWHM (nm)	PLQY (%)	CIE (x, y)
PTPEBP	320	336	532	110	8.14	(0.17, 0.80)

**Table 3:** TNP spike recovery test data in the actual water samples.

	Water Sample	Spike (mg/L)	Found (mg/L)	RSD (%)	Recovery (%)
PTPEBP	Deionized Water	5.00	4.90 ± 0.10	2.04	98.00
	Mineral Water	5.00	4.97 ± 0.08	1.61	99.37
	Tap Water	5.00	5.07 ± 0.11	2.18	101.32

**Table 4:** *t*-test data of fluorescence sensing based on PTPEBP nanoparticles.

	S	$x_{R}$	$\bar{x}$	$t_{exp.}=\frac{\left|x_{R}-\bar{x}\right|}{S/\sqrt{n}}$	t_ref._	Result
Deionized Water	0.10	5.00	4.90	1.73	4.30	1.73 < t_ref._ (acceptable)
Mineral Water	0.08	5.00	4.97	0.65	4.30	0.65 < t_ref._ (acceptable)
Tap Water	0.11	5.00	5.07	1.10	4.30	1.10 < t_ref._ (acceptable)

## Data Availability

Not applicable.

## Supplementary Materials

The following supporting information can be downloaded at: https://www.mdpi.com/article/10.3390/ma16196458/s1.
