# Supplementary material for: Tetraphenylethene-Based Cross-Linked Conjugated Polymer Nanoparticles for Efficient Detection of 2,4,6-Trinitrophenol in Aqueous Phase
Source: Materials (Basel). 2023 Sep 28;16(19):6458. doi: 10.3390/ma16196458 (PMC10573890; doi:10.3390/ma16196458)
Supplement: Supplementary file 1 [file materials-16-06458-s001.zip › materials-2588604-supplementary.pdf]

Supplementary Materials

Tetraphenylethene-Based Cross-Linked Conjugated Polymer Nanoparticles for Efficient Detection of 2,4,6-Trinitrophenol in Aqueous Phase

Shengjie Li [1], Tianwen Ouyang [1], Xue Guo [1], Wenyue Dong [1,2,*], Zhihua Ma [2] and Teng Fei [3,*]

1 School of Materials Science and Engineering, Changchun University of Science and Technology, Changchun 130022, China
2 Chongqing Research Institute, Changchun University of Science and Technology, Chongqing 401135, China
3 State Key Laboratory of Integrated Optoelectronics, College of Electronic Science and Engineering, Jilin University, Changchun 130012, China
* Correspondence: dongwenyue@cust.edu.cn (W.D.); feiteng@jlu.edu.cn (T.F.)

Reagents and Measurements

All reagents were purchased from Energy Chemical and used as received, and the details of reagents are listed in Table S1. Deionized water was used throughout the experiment.

Table S1. Details for reagents.

Reagent	CAS number	Purity
Sodium dodecyl sulfate (SDS)	151-21-3	95%
Potassium carbonate (K_2CO_3)	584-08-7	99%
[1,1'-Biphenyl]-4,4'-diyldiboronic acid	4151-80-8	97%
Tetrakis(triphenylphosphine)palladium ($Pd(PPh_3)_4$)	14221-01-3	99%

The Fourier transform infrared spectroscopy (FT-IR) were obtained on a WQF-510A FT-IR spectrometer (PerkinElmer, USA) with KBr pellet as the reference. ^{1}H NMR spectra were measured on AVANCZ 500 and Bruker AV 600 spectrometers (Bruker, Germany) at 298 K with tetramethylsilane as standard in $CDCl_3$. Nitrogen isotherms were obtained at 77 K on a Tri-star 3000 analyzer (Micromeritics, China). The sample was put into a drying oven and heated at 150 °C for 12 hours, then the sample was placed into the instrument and vacuumed. UV-Visible (UV-Vis) absorption spectra were recorded on a UV-3100 spectrophotometer (SHIMADZU, Japan). Fluorescence emission and excitation spectra were carried out with a RF-5301PC fluorometer (PerkinElmer, USA). The quartz cuvette used in the UV-Vis absorption, fluorescence emission and excitation measurements is 45×12.5×12.5 mm with the capacity of 3500 μL. Samples for UV-Vis, fluorescence emission and excitation spectra were dispersed in deionized water, and 3 mL of the dispersion was added into the cuvette, then the cuvette was put into the liquid tank and measured with the spectrometer. The test range is 400–800 nm for emission spectrum and 200–600 nm for excitation spectrum. The slit is 6–15 nm and the scanning speed is 300 nm/min. The scanning electron microscope (SEM) image was obtained on the JEM-6701 F (JEOL, Japan). The sample was prepared as follows: 2 mg sample was dispersed in 5 mL deionized water and sonicated for 20 minutes; then a drop of the dispersion was placed on a silicon wafer and dried at room temperature. Cyclic voltammetry (CV) was performed by using a standard one-compartment, three-electrode electrochemical cell given by a 100 B/W electrochemical analyzer (BAS, USA), with acetonitrile as solvent and 0.1 M tetrabutylammonium hexafluorophosphate ($TBAPF_6$) as supporting electrolyte. The glassy carbon electrode (GCE) was used as working electrode (WE), platinum (Pt) wire as counter electrode (CE), and Ag/AgCl electrode as reference electrode (RE) standardized against ferrocene/ferrocenium (0.1 M). The potential range is 0–1.4 V, the step potential is 0.001 V and the scan rate is 0.1 V/s. The molecular weight was calculated by static light scattering (SLS) on a DAWN HELEOS–II (Wyatt Technology, USA) at 35 °C. The mobile phase used in SLS

is tetrahydrofuran and the flow rate is 1 mL/min. Polystyrene was used as standard sample in the SLS test.

Table S2. The molecular weight of PTPEBP by SLS.

	M_n (g/mol)	M_w (g/mol)	PDI
PTPEBP	6.618×10^4	2.130×10^5	3.218

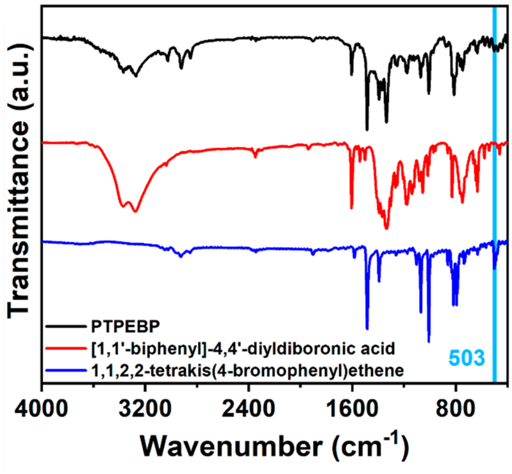

Figure S1. FT-IR spectra of PTPEBP and the corresponding monomers.

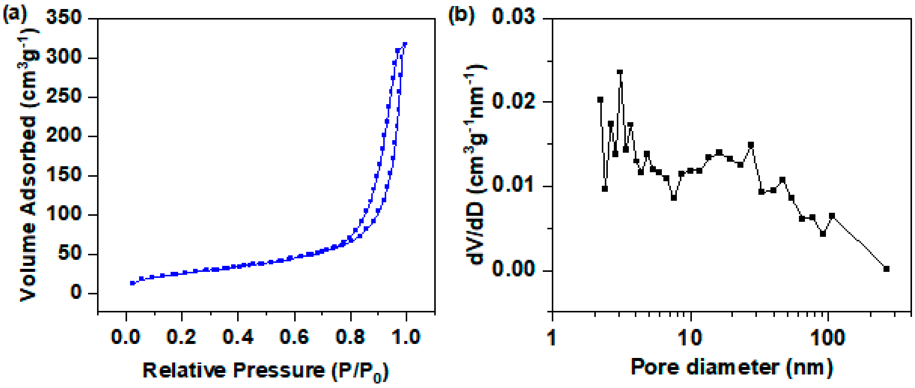

Figure S2. (a) N_2 adsorption/desorption isotherm curve and (b) pore size distribution of PTPEBP nanoparticles.

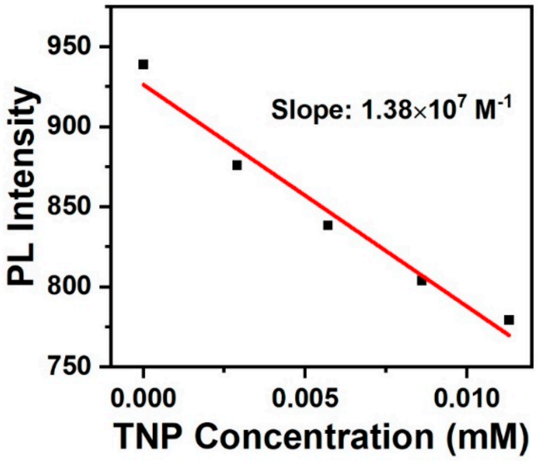

Figure S3. The linear fitting curve of the fluorescence intensity of PTPEBP nanoparticles versus the concentration of TNP.

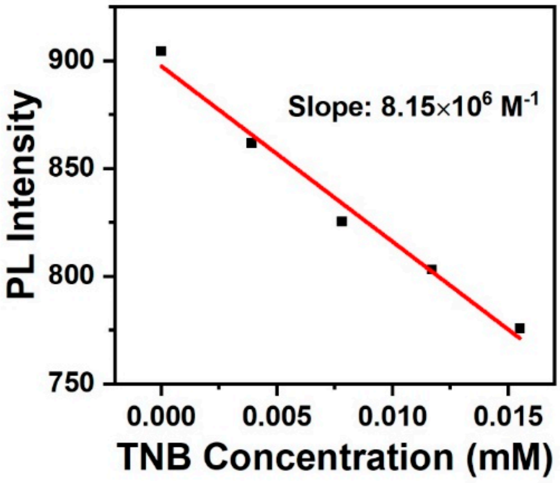

Figure S4. The linear fitting curve of the fluorescence intensity of PTPEBP nanoparticles versus the concentration of TNB.

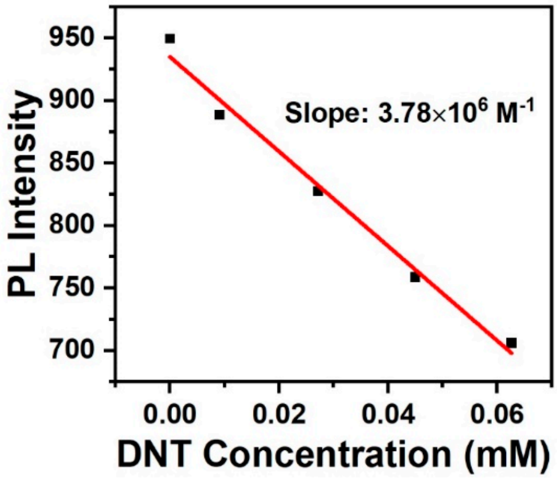

Figure S5. The linear fitting curve of the fluorescence intensity of PTPEBP nanoparticles versus the concentration of DNT.

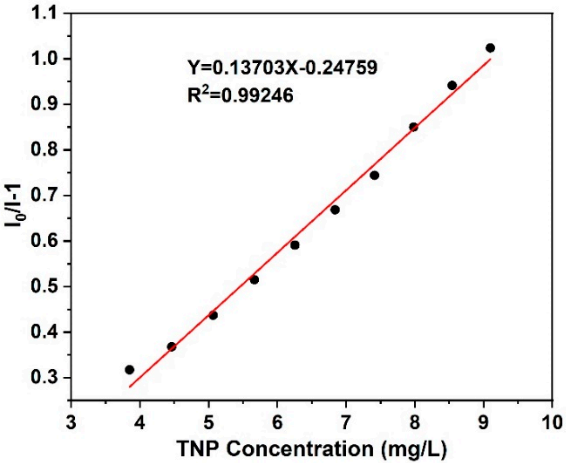

Figure S6. The linear fitting curve of the relative fluorescence intensity of PTPEBP versus the concentration of TNP.

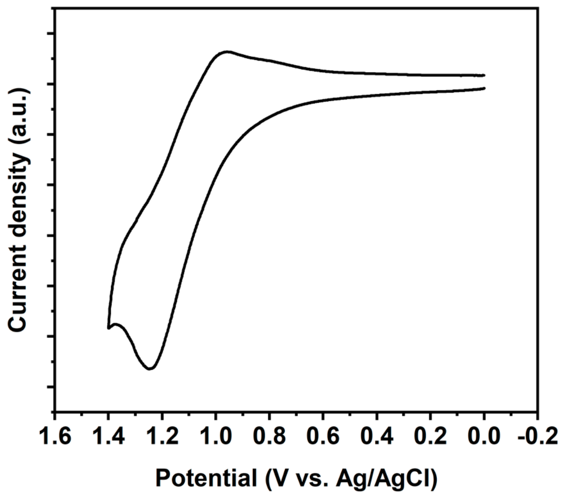

Figure S7. Cyclic voltammogram curve of PTPEBP nanoparticles in acetonitrile (WE: GCE, CE: Pt, RE: Ag/AgCl, potential range: 0–1.4 V, scan rate: 0.1 V/s, step value: 0.001 V, electrolyte: 0.1 M $TBAPF_6$).
